# Accelerating therapeutics development during a pandemic: population pharmacokinetics of the long-acting antibody combination AZD7442 (tixagevimab/cilgavimab) in the prophylaxis and treatment of COVID-19

**DOI:** 10.1128/aac.01587-23

**Published:** 2024-03-27

**Authors:** Lindsay E. Clegg, Oleg Stepanov, Henning Schmidt, Weifeng Tang, Huixia Zhang, Chris Webber, Taylor S. Cohen, Mark T. Esser, Mats Någård

**Affiliations:** 1 Clinical Pharmacology and Quantitative Pharmacology, Clinical Pharmacology & Safety Sciences, R&D, AstraZeneca, Gaithersburg, Maryland, USA; 2 Clinical Pharmacology and Quantitative Pharmacology, Clinical Pharmacology & Safety Sciences, R&D, AstraZeneca, Cambridge, United Kingdom; 3 IntiQuan GmBH, Basel, Switzerland; 4 Clinical Development, Vaccines and Immune Therapies, BioPharmaceuticals R&D, AstraZeneca, Cambridge, United Kingdom; 5 Vaccines & Immune Therapies, BioPharmaceuticals R&D, AstraZeneca, Gaithersburg, Maryland, USA; IrsiCaixa Institut de Recerca de la Sida, Barcelona, Spain

**Keywords:** population pharmacokinetics, SARS-CoV-2, COVID-19, cilgavimab, tixagevimab, AZD7442, monoclonal antibodies

## Abstract

AZD7442 is a combination of severe acute respiratory syndrome coronavirus 2 (SARS-CoV-2)-neutralizing antibodies, tixagevimab and cilgavimab, developed for pre-exposure prophylaxis (PrEP) and treatment of coronavirus disease 2019 (COVID-19). Using data from eight clinical trials, we describe a population pharmacokinetic (popPK) model of AZD7442 and show how modeling of “interim” data accelerated decision-making during the COVID-19 pandemic. The final model was a two-compartmental distribution model with first-order absorption and elimination, including standard allometric exponents for the effect of body weight on clearance and volume. Other covariates included were as follows: sex, age >65 years, body mass index ≥30 kg/m^2^, and diabetes on absorption rate; diabetes on clearance; Black race on central volume; and intramuscular (IM) injection site on bioavailability. Simulations indicated that IM injection site and body weight had > 20% effects on AZD7442 exposure, but no covariates were considered to have a clinically relevant impact requiring dose adjustment. The pharmacokinetics of AZD7442, cilgavimab, and tixagevimab were comparable and followed linear kinetics with extended half-lives (median 78.6 days for AZD7442), affording prolonged protection against susceptible SARS-CoV-2 variants. Comparison of popPK simulations based on “interim data” with a target concentration based on 80% viral inhibition and assuming 1.81% partitioning into the nasal lining fluid supported a decision to double the PrEP dosage from 300 mg to 600 mg to prolong protection against Omicron variants. Serum AZD7442 concentrations in adolescents weighing 40–95 kg were predicted to be only marginally different from those observed in adults, supporting authorization for use in adolescents before clinical data were available. In these cases, popPK modeling enabled accelerated clinical decision-making.

## INTRODUCTION

While the World Health Organization no longer considers coronavirus 2019 (COVID-19) a global health emergency (1), several groups, including immunocompromised individuals, remain at high risk of severe disease, hospitalization, and death due to COVID-19 compared with the general population (2–4). Monoclonal antibodies (mAbs) capable of neutralizing severe acute respiratory syndrome coronavirus 2 (SARS-CoV-2) can protect against COVID-19 and have played a key role in protecting and treating these groups during the COVID-19 pandemic.

AZD7442 comprises two SARS-CoV-2-neutralizing mAbs, tixagevimab and cilgavimab, which are derived from B cells isolated from individuals with prior SARS-CoV-2 infection (5, 6). The fragment crystallizable (Fc) regions of the progenitor mAbs were enhanced with YTE and TM amino acid modifications to extend their serum half-lives (7, 8) and reduce Fc gamma receptor and complement binding (9), respectively. Tixagevimab and cilgavimab prevent viral entry into host cells by simultaneously binding to distinct, non-overlapping epitopes on the SARS-CoV-2 spike protein receptor-binding domain and blocking attachment to human angiotensin-converting enzyme 2 receptors (5, 6). Based on primary results from Phase III studies initiated before the emergence of Omicron variants (10, 11), AZD7442 was authorized for the pre-exposure prophylaxis (PrEP) of COVID-19 in adults and adolescents aged ≥12 years and weighing ≥40 kg in the United States (12), the EU (13), Japan (14), Canada (15), and Australia (16), as well as for the treatment of COVID-19 in the EU and other markets (13–16). Real-world data show that AZD7442 is effective at preventing COVID-19 caused by Omicron BA.1, BA.2, BA.4, and BA.5 variants in immunocompromised individuals (17). However, the Omicron BQ1.1 and XBB sub-variants have demonstrated no *in vitro* susceptibility to neutralization by AZD7442 (18–20) and authorization for use in the United States has been suspended as a result (21).

Population pharmacokinetic (PK) modeling and simulation can accelerate drug development by predicting serum drug concentrations relative to target exposures in novel populations and for novel dosing regimens. This is of special utility in situations when speed is essential, such as pandemics with a rapidly evolving virus (22). Population PK modeling of AZD7442 in adults was first performed using interim PK data from the Phase III PROVENT, STORM CHASER, and TACKLE studies (10, 11, 23) and final data from the first-in-human Phase I study of AZD7442 (24). The results of this “interim” model supported authorization for use in adolescents before clinical data were available and doubling of the recommended dosage to prolong protection against Omicron variants. Here, we describe the final population PK model of AZD7442 in adults, which includes 15 months of follow-up data from the completed Phase III studies of AZD7442, and data from three Phase I studies and two Phase II studies. The impact of covariates on the final model is described, and practical applications of the model are illustrated through examples of its utility during the COVID-19 pandemic.

## RESULTS

### Population analyzed

The final population PK model includes 31,895 serum PK observations from 4,940 participants weighing 36–216 kg from North America, South America, Europe, and Asia. Of these participants, 342 received intravenous (IV) infusions of AZD7442, and 4,598 received intramuscular (IM) injections of AZD7442. Baseline demographics of the combined data set are presented in Table S1.

### Graphical exploration of PK data

**Fig 1:**
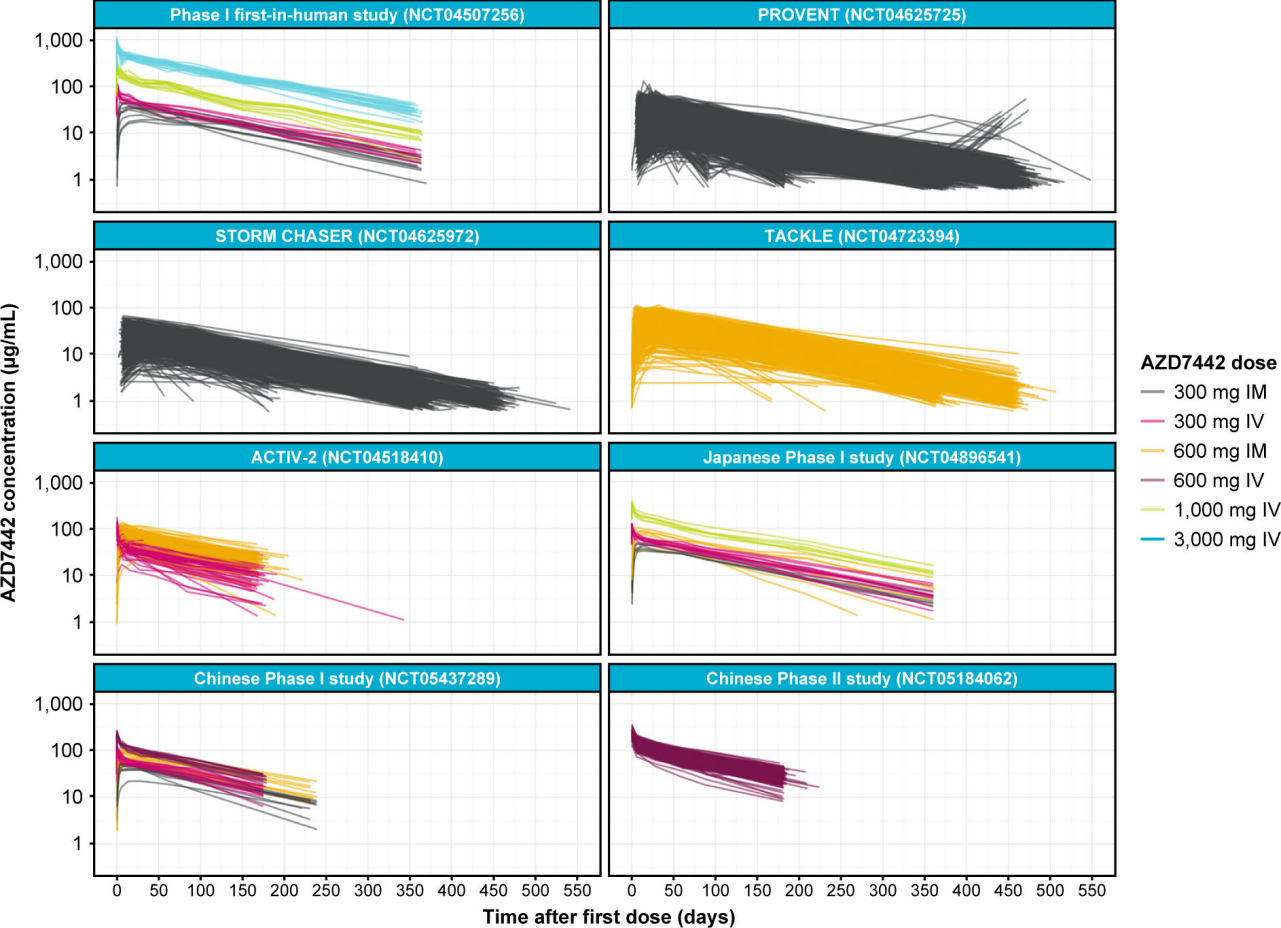
Overview of observed AZD7442 pharmacokinetic data, stratified by study. Spaghetti plots of quantifiable observed AZD7442 data used for model building, colored by dose and route of administration. As summarized in the Materials and Methods section, the following participants were excluded: two in the ACTIV-2 study who received IM injections of AZD7442 *via* the wrong site; 170 without any observation records; and 45 for whom all observations were BLQ or were missing. In addition, implausibly high concentrations of AZD7442 (>100 µg/mL) from 13 participants in the STORM CHASER study and physiologically implausible intermittent BLQ observations were excluded. Note that in PROVENT, some participants received a second dose of AZD7442 before the end of the study follow-up. BLQ, below the limit of quantitation; IM, intramuscular; IV, intravenous.

PK data stratified by study are shown in Fig. 1. The observed serum concentration-time curves following IV administration suggest a biphasic distribution and elimination profile, thereby supporting the use of a two-compartmental distribution model to characterize the dose-concentration relationship of AZD7442.

**Fig 2:**
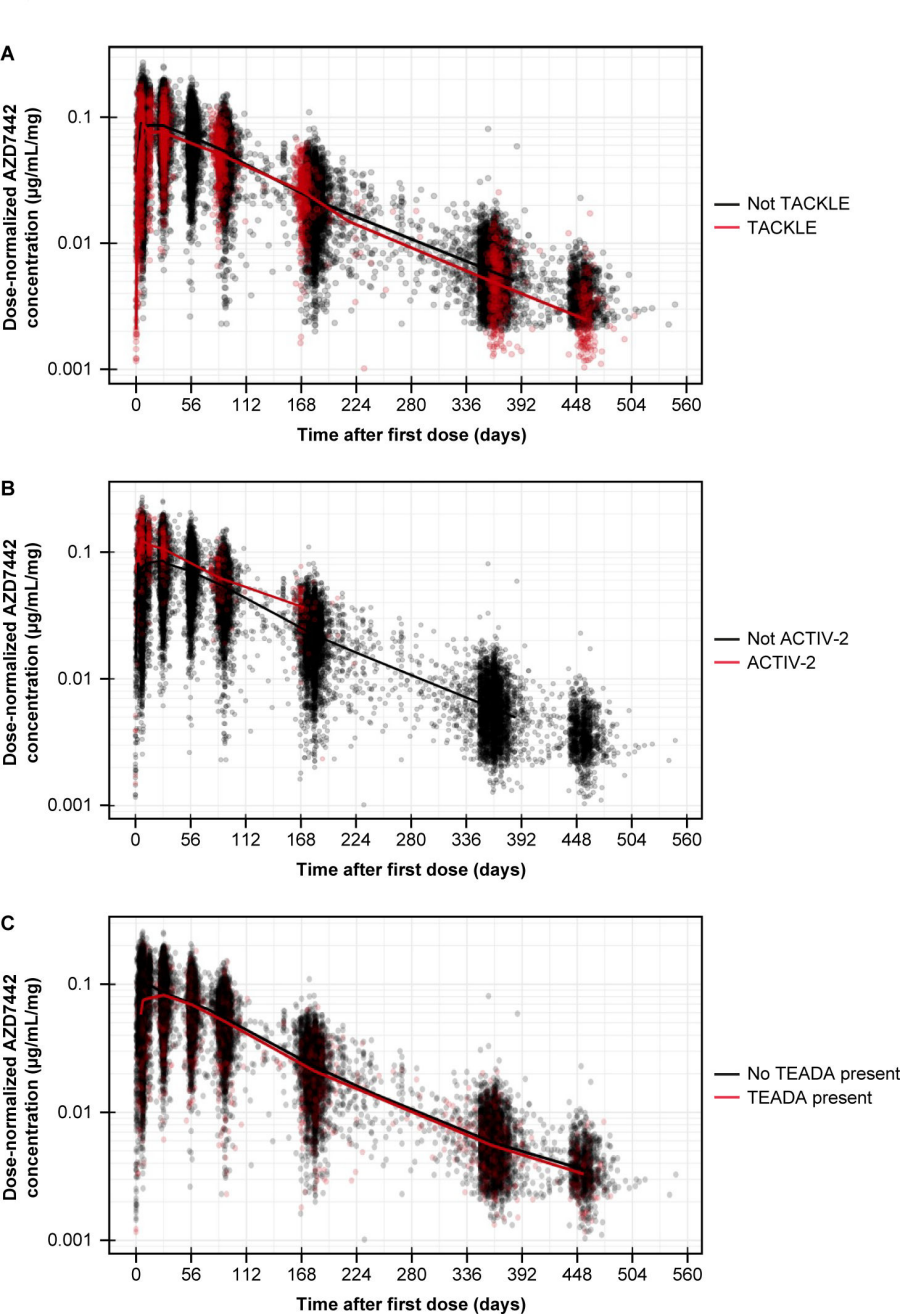
Comparison of serum AZD7442 concentrations in (**A**) prophylactic versus treatment settings, (**B**) by injection site, and (**C**) by presence of TEADA. (**A**) Comparison of dose-normalized IM AZD7442 PK profiles between TACKLE (treatment) and other studies considering prophylaxis. (**B**) Comparison of dose-normalized IM AZD7442 PK profiles between ACTIV-2 (thigh) and non-ACTIV2 (gluteal) studies. (**C**) Comparison of dose-normalized IM AZD7442 PK profiles between participants with negative and positive TEADA status. TEADA positive was defined as either ADA negative at baseline and ADA positive at ≥1 day post-baseline visit, or baseline ADA positive that was boosted to ≥4 fold during the study. Conversely, "No TEADA" included participants who were ADA negative and those ADA-positive participants who did not meet the definition of TEADA. Participants with missing TEADA status were excluded. For all panels, dots indicate individual serum concentrations, lines are median profiles, and BLQ data are not shown. ADA, antidrug antibodies; BLQ, below the limit of quantitation; IM, intramuscular; IV, intravenous; PK, pharmacokinetic; TEADA, treatment-emergent antidrug antibodies.

Whether the PK of AZD7442 differed between prophylaxis and treatment settings of COVID-19 was assessed first. Dose-normalized PK profiles of AZD7442 following IM injection into the gluteal region were compared between TACKLE (treatment setting) and other included studies (except ACTIV-2, during which AZD7442 was injected into the anterolateral thigh) (Fig. 2A). No difference in AZD7442 PK was observed between the treatment setting of TACKLE and the prophylaxis setting of other studies. Whether the PK of AZD7442 differed depending on the site of IM injection was assessed next. Dose-normalized PK profiles of AZD7442 were compared between the ACTIV-2 study, during which AZD7442 was injected into the anterolateral thigh, and other included studies, during which AZD7442 was injected into the gluteal region (Fig. 2B). Injection into the thigh appeared to result in faster absorption and higher bioavailability compared with injection into the gluteal area, prompting further investigation *via* covariate modeling. Finally, it was assessed whether the PK of AZD7442 was impacted by treatment-emergent antidrug antibodies (TEADAs). Dose-normalized profiles of AZD7442 were compared between participants with and without TEADAs (Fig. 2C), with TEADAs found to have no notable impact on AZD7442 PK.

### Model building and evaluation

Following graphical exploration of the PK data, a base model for AZD7442 was developed, which was further updated following covariate testing. The AZD7442 base model structure was similar to that used for the “interim” model, with the effect of body weight on clearance (CL), central volume of distribution (Vc), inter-compartmental clearance (Q), and peripheral volume of distribution (Vp) considered a covariate with fixed allometric scaling (Table S2). The base model was fit to log-transformed serum concentrations, and included separate additive residual error models for IV and IM data, as well as the correlation between CL and Vc; first-order absorption rate constant (ka) and Vc; and ka and CL. Body weight was confirmed as having a statistically significant effect on clearance and volume (central and peripheral). Other covariates meeting the pre-specified effect size required for inclusion in the final model (Table S2) were as follows: male sex (72.1% faster absorption versus females); age >65 years (27.7% slower absorption versus age ≤65 years); diabetes (27.2% slower absorption and 21.9% higher CL versus non-diabetes); Black race (22.2% lower Vc versus non-Black race); body mass index (BMI) ≥30 kg/m^2^ (22.4% slower absorption versus BMI <30 kg/m^2^); and IM injection into the thigh (51.6% higher bioavailability versus the gluteal region). The effect of the IM injection site on absorption observed in Fig. 2C did not meet the pre-defined criteria for inclusion. TEADAs were not found to have a potentially clinically relevant impact on CL, and no impact of renal function (based on baseline estimated glomerular filtration rate) or any of the hepatic function markers on CL were identified; these covariates were therefore not included in the final model.

The parameter estimates for the final model of AZD7442 are reported in Table 1 and Table S3. The final AZD7442 model was evaluated on both cilgavimab and tixagevimab data sets, and a comparison of parameter estimates is provided in Table 1 and Tables S3 to S5. All PK parameters in all three models were estimated with good precision and were very similar for AZD7442, cilgavimab, and tixagevimab, confirming similar PK for these YTE-modified mAbs. For the AZD7442 model, the relative standard error (RSE) for typical (population) parameters and random effects was <5.1%. All covariate coefficients had an RSE of <16.3%. Inter-individual variability (percent coefficient of variation) of AZD7442 was 42.6% for CL, 39.8% for Vc, 35.9% for Vp, and 59.9% for ka. Terminal elimination half-lives of AZD7442, tixagevimab, and cilgavimab were estimated based on observed concentration data using micro-constants. The median terminal elimination half-lives of AZD7442, tixagevimab, and cilgavimab were estimated to be 78.6, 81.3, and 78.0 days, respectively (Table 2).

**TABLE 1:** Population PK parameter estimates (95% CI) for AZD7442, cilgavimab, and tixagevimab^*a*^

Parameter	Units	AZD7442	Cilgavimab	Tixagevimab
Typical parameters				
First-order absorption rate (ka)	1/day	0.11 (0.104, 0.115)	0.119 (0.113, 0.126)	0.122 (0.115, 0.129)
Clearance (CL)	L/day	0.0504 (0.0486, 0.0522)	0.0516 (0.0492, 0.054)	0.0457 (0.0441, 0.0473)
Central volume of distribution (Vc)	L	3.36 (3.21, 3.52)	3.52 (3.28, 3.76)	3.17 (3.04, 3.31)
Absolute IM bioavailability (FIM)	Fraction	0.671 (0.649, 0.693)	0.658 (0.629, 0.687)	0.615 (0.595, 0.635)
Inter-compartmental clearance (Q)	L/day	0.395 (0.382, 0.408)	0.485 (0.462, 0.509)	0.432 (0.416, 0.449)
Peripheral volume of distribution (Vp)	L	1.83 (1.76, 1.9)	1.82 (1.76, 1.88)	1.77 (1.69, 1.85)
Inter-individual variability				
ka	%CV	59.9 (58, 61.8)	77.1 (75.1, 79.1)	78.6 (76.3, 80.8)
CL	%CV	42.6 (41.8, 43.5)	44.1 (43.3, 44.9)	40.8 (40, 41.6)
Vc	%CV	39.8 (38.3, 41.3)	52.2 (50.3, 54)	38 (36.5, 39.6)
FIM	%CV	0	0	0
Q	%CV	0	0	0
Vp	%CV	35.9 (33.7, 38.1)	24.5 (22, 25)	36.8 (34.7, 39.1)
Correlation of random effects				
(ka, CL)	−	–0.387 (–0.418, –0.356)	–0.442 (–0.469, –0.415)	–0.496 (–0.526, –0.466)
(ka, Vc)	−	–0.689 (–0.728, –0.651)	–0.546 (–0.577, –0.516)	–0.835 (–0.878, –0.791)
(CL, Vc)	−	0.588 (0.566, 0.611)	0.764 (0.75, 0.778)	0.697 (0.679, 0.715)
Parameter-covariate relationships				
beta_ka(SEXM_1)	−	0.543 (0.494, 0.591)	0.57 (0.517, 0.624)	0.632 (0.574, 0.689)
beta_ka(AGECAT_1)	−	–0.325 (–0.387, –0.263)	–0.392 (–0.459, –0.324)	–0.443 (–0.515, –0.371)
beta_ka(BMICAT_1)	−	–0.254 (–0.304, –0.205)	–0.188 (–0.242, –0.133)	–0.217 (–0.276, –0.158)
beta_ka(DIAB_1)	−	–0.318 (–0.388, –0.249)	–0.27 (–0.347, –0.192)	–0.262 (–0.345, –0.178)
beta_CL(BWT)	−	0.75 (NA, NA)	0.75 (NA, NA)	0.75 (–, –)
beta_CL(DIAB_1)	−	0.198 (0.166, 0.229)	0.149 (0.122, 0.176)	0.167 (0.138, 0.196)
beta_Vc(BWT)	−	1 (NA, NA)	1 (NA, NA)	1 (–, –)
beta_Vc(RACEB_1)	−	–0.251 (–0.285, –0.217)	–0.26 (–0.29, –0.23)	–0.242 (–0.274, –0.21)
beta_Q(BWT)	−	0.75 (NA, NA)	0.75 (NA, NA)	0.75 (–, –)
beta_Vp(BWT)	−	1 (NA, NA)	1 (NA, NA)	1 (–, –)
beta_FIM(ACTIV2_1)	−	0.416 (0.359, 0.474)	0.414 (0.345, 0.482)	0.378 (0.319, 0.437)
Residual variability				
error_ADD1 (IM data)	−	0.24 (0.239, 0.241)	0.24 (0.239, 0.24)	0.272 (0.271, 0.273)
error_ADD2 (IV data)	−	0.104 (0.103, 0.104)	0.119 (0.118, 0.12)	0.108 (0.107, 0.108)

^*a*^ BMI, body mass index; BWT, body weight; CAT, category; CL, clearance; %CV, percent coefficient of variation; DIAB, diabetes; FIM, absolute IM bioavailability; IM, intramuscular; IIV, inter-individual variability; IV, intravenous; ka, first-order absorption rate constant; NA, not available; PK, pharmacokinetic; Q, inter-compartmental clearance; RACEB, race: Black; SEXM, sex: male; Vc, central volume of distribution; Vp, peripheral volume of distribution.

**TABLE 2:** Terminal elimination half-lives estimated for AZD7442, cilgavimab, and tixagevimab

Analyte	Median (5th–95th percentiles), days
AZD7442	78.6 (45.6–101.0)
Tixagevimab	81.3 (49.3–106.0)
Cilgavimab	78.0 (49.2–97.4)

**Fig 3:**
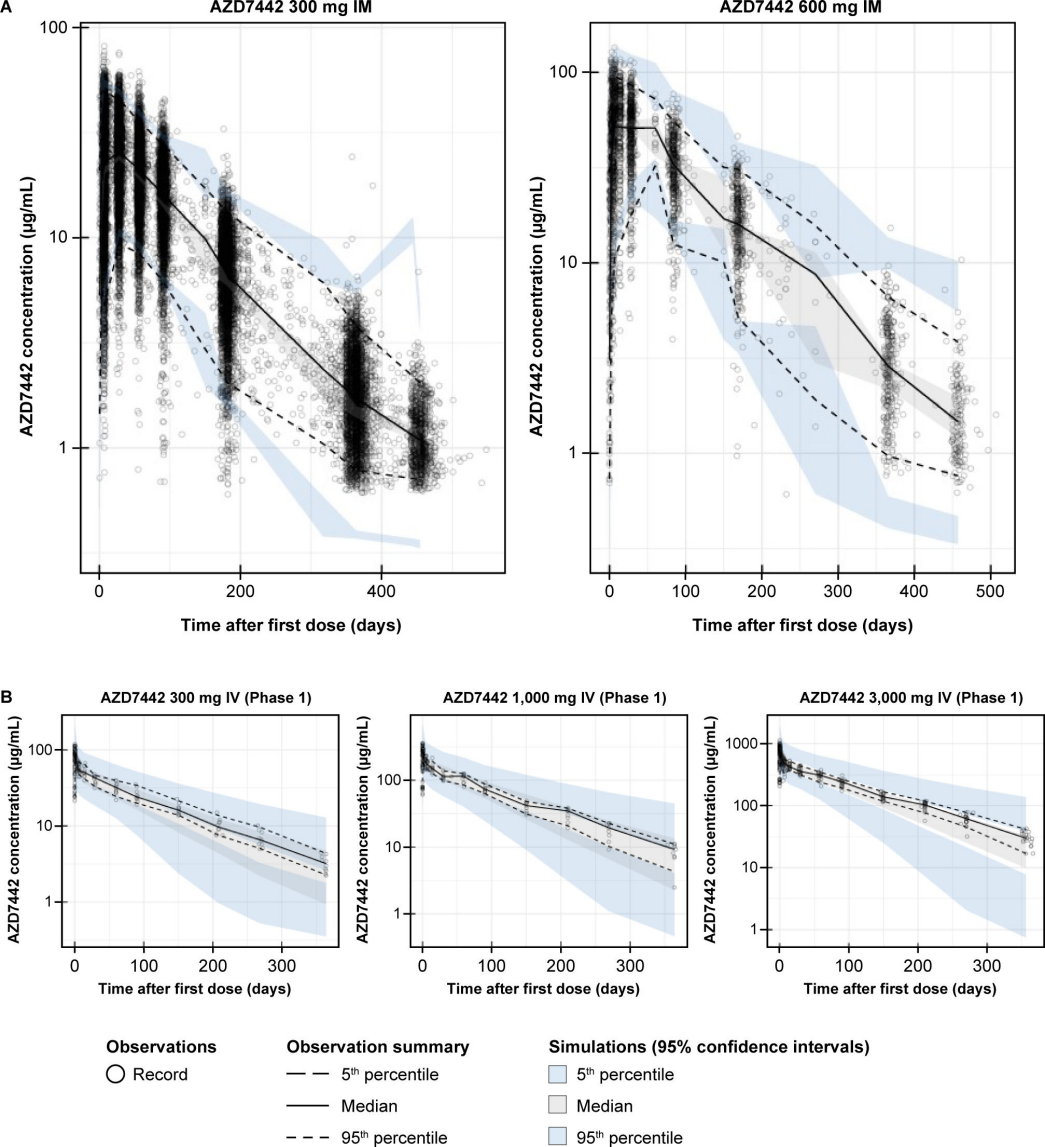
Selected visual predictive checks for the final AZD7442 model, stratified by (**A**) IM dose levels and (**B**) IV dose levels in the Phase I first-in-human study. The open circles represent the observed PK data from the pooled analysis. The black line represents the median of the observed PK data, and the upper and lower dashed lines are the 95th and 5th percentiles. The gray-shaded area represents the median of the predicted data from the AZD7442 model, and the upper and lower blue-shaded areas represent the 95th and 5th percentiles. IM, intramuscular; IV, intravenous; PK, pharmacokinetic; VPC, visual predictive check.

The predictive performance of the final AZD7442 PK model was assessed using visual predictive checks (VPCs) and goodness-of-fit plots (Fig. S1). VPCs stratified by study and dose compared observed and predicted exposures of AZD7442 in adults (Fig. 3). The resulting plots suggest there is adequate agreement between observed and simulated statistics from the final AZD7442 population PK model. Therefore, the final model can be used for simulations. The predictive performance of the final PK models for cilgavimab and tixagevimab was similarly assessed using VPCs (Fig. S2) . The results suggest a similar agreement between the observed and simulated statistics, as observed for the final PK model of AZD7442.

### Simulation-based analyses

**Fig 4:**
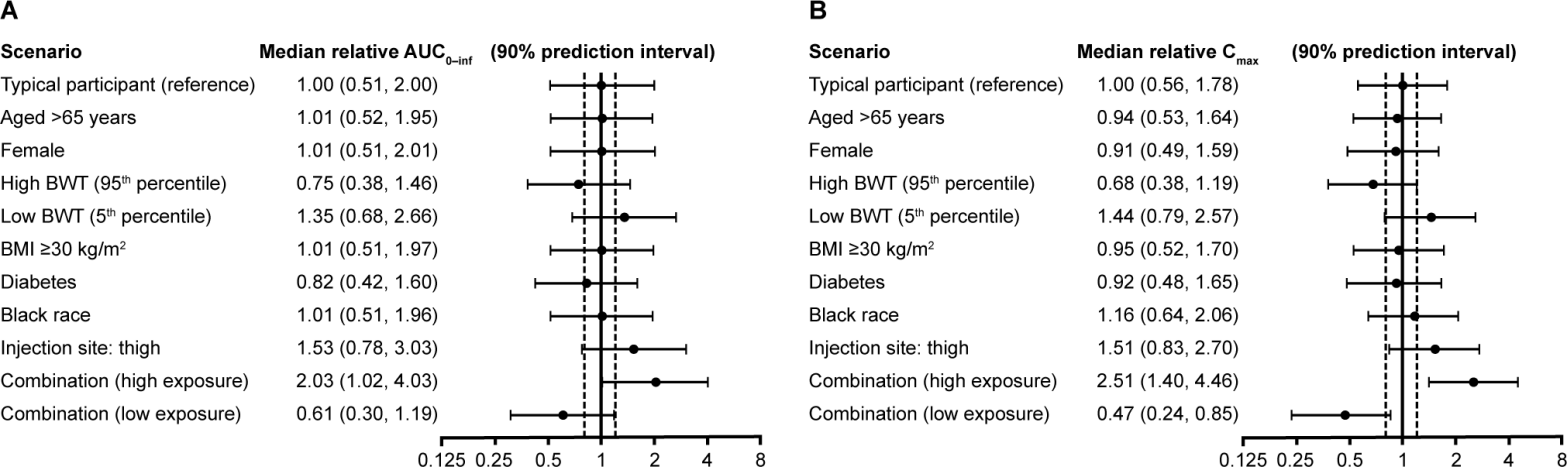
(**A**) Relative AUC_0-inf_ and (**B**) relative C_max_ by covariate scenario following a single intramuscular dose of AZD7442 600 mg. In each covariate scenario, 10,000 individual sets of pharmacokinetic parameters were drawn from the variability distribution, based on which predictions for the exposure metrics were simulated. Relative exposure metrics (median and 90% prediction interval) were calculated for each covariate scenario, relative to the median of the exposure metric for the typical subject. Typical subject (comparator group): BWT 80.6 kg, male, age ≤65 years, BMI < 30 kg/m^2^, non-Black race, no diabetes, gluteal injection site. Combination: high (combines the factors predisposing to high exposure): BWT 55.6 kg, male, age ≤65 years, BMI < 30 kg/m^2^, Black race, no diabetes, thigh injection site. Combination: low (combines the factors predisposing to low exposure): BWT 122 kg, female, age >65 years, BMI ≥ 30 kg/m^2^, non-Black race, diabetes, gluteal injection site. The dotted lines represent a 20% effect size. AUC_0-inf_, area under the concentration-time curve from time 0 to infinity; BMI, body mass index; BWT, body weight; C_max_, maximum serum concentration.

The potential clinical relevance of covariates retained in the final AZD7442 model was assessed using a simulation approach, whereby exposure metrics were derived for individual covariates and covariate combinations of interest. Based on simulations, body weight and IM injection site were the two covariates found to have a > 20% predicted effect on exposure (Fig. 4). Compared with a typical participant, maximum serum concentration (C_max_) was 44% higher and area under the concentration-time curve from time 0 to infinity (AUC_0-Inf_) was 35% higher for the 5th percentile of body weight; C_max_ was 32% lower and AUC_0-Inf_ 25% was lower for the 95th percentile of body weight. Compared with IM injection into the gluteal region, C_max_ and AUC_0-Inf_ were predicted to be 51% and 53% higher, respectively, for injection into the thigh. None of the retained covariates, including age, were considered to have a clinically relevant impact requiring a dose adjustment.

### Applications

Here, we describe two examples of how an “interim” population PK model facilitated rapid decision-making with respect to PrEP with AZD7442 during the fast-moving COVID-19 pandemic. For consistency, we present results from the final population PK model here, which lead to the same conclusions as the results of the “interim” model.

#### Inclusion of adolescents in the AZD7442 indication

**Fig 5:**
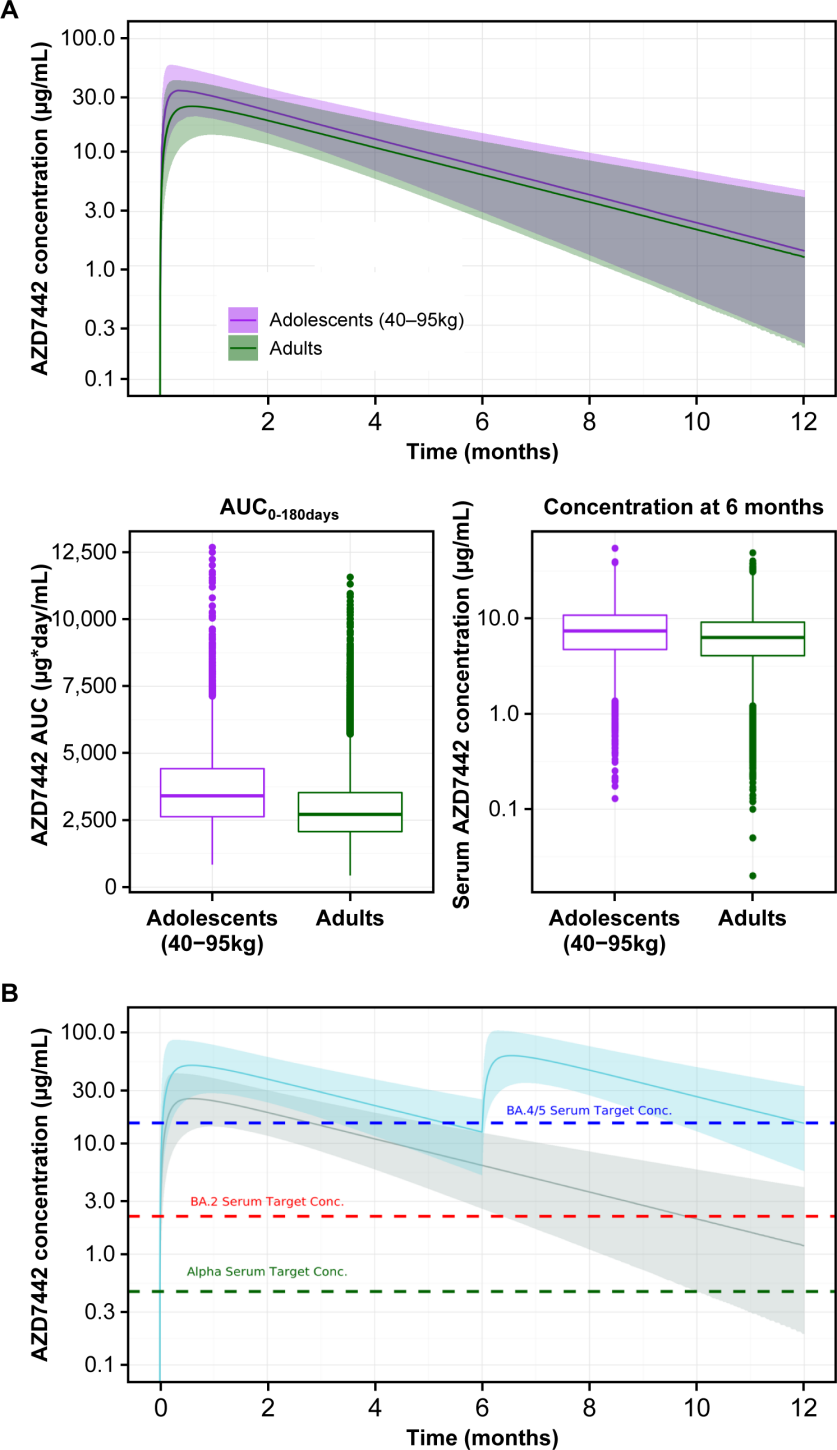
(**A**) PK extrapolation from adults to adolescents weighing 40–95 kg based on AZD7442 exposure-matching and (**B**) Population PK-based justification for the change in AZD7442 pre-exposure prophylaxis posology from 300 mg IM to 600 mg IM every 6 months in response to emerging Omicron variants of concern. (**A**) Comparison of simulated AZD7442 serum concentrations over time for adolescents with the weight range of 40 to 95 kg and adults (weight distribution matched to the population PK data set). The solid line represents a median predicted concentration; the ribbon represents an 80% prediction interval. (**B**) Predicted duration of protection for a single dose of AZD7442 300 mg (gray) and AZD7442 600 mg every 6 months (blue) against selected SARS-CoV-2 variants of concern (dashed lines). The solid lines represent the median predicted AZD7442 serum concentration, with the ribbon representing the 80% prediction intervals. Dashed horizontal lines = serum target concentration for Alpha, BA.2, and BA.4/5 calculated using IC50 from pseudotyped virus-like particle assay and upper respiratory tract partition ratio of 1.81%. Serum target for 80% inhibition in nasal lining fluid (prophylaxis) = IC80/partitioning ratio = (IC50 × 4)/partitioning ratio assuming a Hill coefficient of 1. IC50 for AZD7442 against the Alpha, Delta, BA.2, and BA.4/5 subvariants = 2.1, 2.2, 9.8, and 69.4 ng/mL (Monogram Biosciences). AUC, area under the curve; Conc., concentration; IC50, 50% maximum inhibitory concentration; IC80, 80% maximal inhibitory concentration; IM, intramuscular; PK, pharmacokinetic.

During the COVID-19 pandemic, studies of AZD7442 only enrolled adults aged 18 years or older. However, allometric scaling of mAb PK is reported to be generally similar between adults and adolescents (25), and age was found not to have a clinically meaningful effect on the final population PK model. Adolescents whose body weights were within the range of adults enrolled in the PROVENT, STORM CHASER, and TACKLE studies (≥40 kg) were therefore expected to have comparable AZD7442 exposure to an adult population. To support the appropriateness of an adult dose of AZD7442 for adolescents weighing ≥40 kg, predicted AZD7442 exposures in adults and adolescents were compared. Figure 5 shows simulations for an adolescent population, assuming a uniform distribution of body weights between 40 and 95 kg, compared with the adult population included in the population PK analysis. Only marginal differences in drug exposure, assessed as AUC_0-180days_ and serum concentrations at 6 months (the intended duration of protection), were predicted, with largely overlapping distributions for adult and adolescent exposures. These differences were not considered to be clinically meaningful. As cilgavimab and tixagevimab have no endogenous targets, comparable exposure between adults and adolescents is expected to result in comparable efficacy and safety. Based on these results, AZD7442 was authorized for use in adolescents before PK, efficacy, and safety data were available from TRUST, an ongoing Phase I study of AZD7442 in pediatric participants (clinicaltrials.gov identifier: NCT05281601).

#### AZD7442 PrEP dosage increase in response to emerging Omicron variants

During 2022, new SARS-CoV-2 Omicron variants emerged that were less susceptible to neutralization by cilgavimab and/or tixagevimab (i.e., with higher *in vitro* half-maximal inhibitory concentrations [IC50s]) (8–10).

For PrEP, the target site of action is assumed to be the nasal lining fluid. The predicted target serum concentration for AZD7442 to be effective against a given variant was derived by assuming that AZD7442 concentrations in the nasal lining fluid corresponding to a neutralization target of 80% would be sufficient for the prevention of symptomatic COVID-19 infection. As the precise level of viral inhibition required for effective prophylaxis was (and remains) unknown, the target of 80% inhibition was selected based on available viral dynamic modeling of SARS-CoV-2 and other viruses (26) to give a reasonable estimate of an AZD7442 concentration expected to be associated with efficacy. Partitioning into the nasal lining fluid was set at 1.81%, based on data from the AZD7442 first-in-human study (6, 24). Population PK simulations suggested that the 300 mg IM dose of AZD7442 would only provide a few months of protection against the BA.2 and BA.4/5 Omicron subvariants while doubling the dose to 600 mg would provide 4–6 months of protection (Fig. 5). Based on this analysis and safety data for the 600 mg IM dose available from the TACKLE study, the recommended AZD7442 dosage for PrEP was updated from 300 mg to 600 mg. Notably, these model predictions are consistent with real-world effectiveness data that have become available recently following the BA.2 and BA.5 waves, showing protection lasting 4–6 months depending on the Omicron variant (27). This analysis also informed the introduction of a 6-month redosing interval ahead of the availability of clinical data on repeat dosing of AZD7442.

## DISCUSSION

Population PK modeling and simulation represent an effective approach for accelerating drug development in populations with high unmet needs when PK is well characterized and decisions need to be made more quickly than clinical data or real-world effectiveness data can be generated. In such cases, population PK modeling is increasingly being used to guide regulatory and labeling decisions before clinical data become available, and in populations or applications where clinical trials are not feasible. A previous population PK analysis performed using interim data from the then-ongoing PROVENT, STORM CHASER, and TACKLE studies and final data from the first-in-human Phase I study indicated that AZD7442, cilgavimab, and tixagevimab had comparable PK and followed linear kinetics, and predicted that AZD7442 exposure would be similar between adults and adolescents with body weights within range of the adults included in the aforementioned Phase III studies (40–95 kg). As tixagevimab and cilgavimab have no endogenous targets, similar AZD7442 exposure was expected to result in comparable efficacy and safety. Consequently, AZD7442 was authorized for use in adolescents before PK data in an adolescent population were available from clinical studies. Simulations then indicated that doubling the recommended PrEP dosage from 300 mg to 600 mg and imposing a 6-month redosing interval, would prolong protection against emerging Omicron variants, which are less susceptible to neutralization by tixagevimab and/or cilgavimab.

The final population PK model described herein includes 15 months of follow-up data from the now completed PROVENT, STORM CHASER, and TACKLE studies (10, 11, 23), as well as additional data from two Phase I studies in healthy Japanese (28) and Chinese participants, one Phase II study in Chinese adults, and the Phase II ACTIV-2 study of outpatients with COVID-19 (29). YTE-modification of the Fc regions of tixagevimab and cilgavimab was intended to prolong the duration of protection by extending their serum half-lives and eliminating the need for regular redosing. This final model confirms that AZD7442, cilgavimab, and tixagevimab have comparable PK, follow linear kinetics, and have extended half-lives (median 78.0–81.3 days) that are consistent with prior observations (24). The PK of AZD7442 is also comparable with the PK reported in adults for another YTE-modified mAb, nirsevimab (30), suggesting consistent and predictable PK across YTE-modified mAbs.

Several covariates were retained in the final model: body weight was included for CL, Vc, Q, and Vp using standard allometric exponents; male sex, age >65 years, and BMI ≥ 30 kg/m^2^ were included for ka, diabetes status was included for ka and CL, Black race was included for Vc, and site of IM injection was included for bioavailability. This final model indicates that the PK of AZD7442 is consistent between prophylaxis and treatment settings of COVID-19 and between participants with or without TEADAs. Body weight was predicted to have a > 20% effect on AZD7442 exposure, as expected for a mAb. IM injection into the thigh resulted in higher bioavailability compared with injection into the gluteal region, with a > 20% effect on AZD7442 exposure. This result, based on data from the ACTIV-2 study, where IM injection of AZD7442 into the thigh achieved more rapid attainment of C_max_ than is observed when AZD7442 is administered in the gluteal region, suggests IM administration in the anterolateral thigh may provide earlier achievement of effective serum concentrations in individuals with SARS‐CoV‐2 infection (29). Limited data are available in the literature on the impact of IM administration sites on absorption and bioavailability, as the majority of therapeutic mAbs are delivered intravenously or subcutaneously. Subcutaneous bioavailability typically ranges from 50% to 80%, with no clear impact of subcutaneous administration site on bioavailability (31, 32). However, bioavailability is typically different for subcutaneous and IM administration, and it is possible that differences in tissue composition in the anterolateral thigh and gluteal region may contribute to the exposure differences observed in this analysis. These data motivate further evaluation of the impact of different IM injection sites on the PK of mAbs targeting SARS-COV-2 and other exogenous targets. However, importantly, our simulation-based assessment suggested that none of the retained covariates, including body weight, injection site, and age would have a clinically relevant impact on PK that would require a dose adjustment. Moreover, serum concentrations of AZD7442 simulated for adolescents weighing 40–95 kg were not meaningfully different from those observed in the adult population. The earlier decision to authorize the use of AZD7442 in adolescents based on the “interim” model is therefore supported, allowing access to AZD7442 for vulnerable adolescents more quickly than would otherwise have been possible.

Another decision facilitated by the “interim” model was the doubling of the recommended PrEP dose from 300 mg to 600 mg, with redosing introduced after 6 months, to counter the emergence of SARS-CoV-2 Omicron variants less susceptible to neutralization by cilgavimab and/or tixagevimab. Clinical studies of new mAbs are slow relative to the speed at which SARS-CoV-2 is evolving. As shown in this case, population PK analyses can facilitate rapid updates of dosing recommendations based on existing clinical data. Using data from this final population PK analysis, simulations were performed with a neutralization target of 80% and assuming 1.81% partition into the nasal lining fluid (the site of initial infection) (6, 24). The results show that a 600 mg PrEP dose of AZD7442 would provide 4–6 months of protection against both BA.2 and BA.4/5 Omicron subvariants, compared with only a few months for the 300 mg dose. The earlier decision to double the dose based on the “interim” model is therefore supported by the final model, as well as real-world effectiveness data (27).

While the suspension of the authorization for use of AZD7442 in the United States (21) limits the direct applicability of the present analysis, the work presented applies to the development of other YTE-modified mAbs targeting SARS-CoV-2 and other exogenous targets, due to the similar PK expected (and observed for tixagevimab and cilgavimab here) for YTE-mAbs. Therefore, this population PK model could be used to more quickly and confidently inform the selection of dosing regimens for future mAbs, including in potential pandemic scenarios with a rapidly evolving virus. An additional limitation of this analysis is the small proportion of immunocompromised individuals included in the final model (<1% of the total population). While this is now a key target population for PrEP of COVID-19, few immunocompromised individuals were enrolled in the AZD7442 studies included in this analysis, precluding detailed evaluation of immunocompromised status as a covariate. While no specific differences in PK of AZD7442 are expected in individuals with immunocompromising conditions, additional data collection among this key population is ongoing for AZD7442 (NCT05375760), as well as for a novel YTE-modified COVID-19 mAb (NCT05648110). As clinical studies have shown that AZD7442 is well tolerated and has a favorable safety profile at doses of 300 to 600 mg administered intramuscularly (10, 11, 23) and up to 3,000 mg administered intravenously (24), we did not assess the impact of exposure on the safety of AZD7442 in this analysis. Finally, the target of 80% inhibition in the nasal lining fluid used to inform the AZD7442 PreP dose increase in response to the emergence of Omicron variants, including BA.2 and BA.5, is based on viral dynamic modeling and theoretical assumptions, as no definitive correlate of protection has been established to relate serum concentrations and efficacy for mAbs that neutralize SARS-CoV-2, although work in this area is emerging (33, 34).

The final population PK model of AZD7442 supports key decisions made during the COVID-19 pandemic based on predictions of PK generated from limited clinical data. In the event of another pandemic or other urgent scenario, population PK analysis could be used to predict PK and accelerate access to new YTE-modified mAbs such as AZD3152, which is currently under development to prevent and treat COVID-19 caused by SARS-CoV-2 subvariants that are resistant to neutralization by AZD7442 (NCT05648110).

## MATERIALS AND METHODS

### Data sets

The final population PK model included serum PK data from the following eight studies of AZD7442 (Table S6): the Phase I first-in-human study in healthy adults (NCT04507256) (24); two Phase I studies in healthy Japanese (NCT04896541) (28) and Chinese (NCT05437289) adults; one Phase II study in Chinese adults (healthy and those with stable medical conditions; NCT05184062); one Phase II study of outpatients with COVID-19 (ACTIV-2; NCT04518410) (29); and the Phase III, randomized, double-blind, placebo-controlled studies of AZD7442 in PrEP (PROVENT; NCT04625725) (10), post-exposure prophylaxis (STORM CHASER; NCT04625972) (23), and treatment (TACKLE; NCT04723394) (11) settings of COVID-19. Participants received IM injections of AZD7442 at doses ranging between 300 and 600 mg, or IV infusion of AZD7442 at doses ranging between 300 and 3,000 mg. IM injections of AZD7442 were administered into the gluteal region except in the ACTIV-2 study, where they were administered into the thigh. All studies involved the administration of a single dose of AZD7442 except for PROVENT, during which some participants received a second dose of AZD7442 after 10–14 months in a sub-study. All studies were conducted in accordance with the ethical principles derived from international guidelines and were approved by an institutional review board or ethics committee.

### PK sampling and assays

PK sampling schemes are summarized in Table S6. Tixagevimab and cilgavimab serum concentrations were measured by PPD Laboratories (Richmond, VA, USA) using validated hybrid ligand binding liquid chromatography with a tandem mass spectrometry assay, which is capable of distinguishing tixagevimab and cilgavimab in human sera based on distinct complementarity-determining region peptide sequences (35). The assay’s lower limit of quantification (LLOQ) was 0.3 µg/mL. AZD7442 concentrations were calculated as summations of tixagevimab and cilgavimab concentrations. During assay validation, inter-assay precision (relative error) ranged between 1.8% and 10.5% for tixagevimab and between 5.1% and 11.1% for cilgavimab. Inter-assay accuracy (standard deviation) ranged between –20.8% and 8.2% for tixagevimab and between –7.6% and 5.3% for cilgavimab.

#### Handling of data exclusions, data below the LLOQ, and outliers

Data pertaining to two participants in the ACTIV-2 study who received IM injection of AZD7442 into the wrong administration site, 170 participants with no observation records, and 45 participants for whom all observations were either below the limit of quantitation (BLQ) or missing, were excluded from the analysis. BLQ data corresponded to 6.2%, 8.0%, 8.5%, and 15.7% of observations in the PROVENT, STORM CHASER, ACTIV-2, and TACKLE studies, respectively; however, when only values between 2 hours and 12 months post-dose were considered, these proportions dropped to <3% for AZD7442, tixagevimab, and cilgavimab. BLQ observations were handled using the “M3” method in the NONMEM nonlinear mixed-effects modeling software (i.e., including the probability that model-predicted concentrations for BLQ observations are below the LLOQ as part of the maximum likelihood estimation), although the “M1” method was also assessed and resulted in similar parameter estimates and covariate effects (36).

In the STORM CHASER study, 13 of 4,922 (0.3%) observations had implausibly high AZD7442 concentrations (>100 µg/mL) and were excluded (although sensitivity analyses show that these values have no impact on the results). Across the PROVENT, STORM CHASER, TACKLE, and ACTIV-2 studies, BLQ observations were identified in between observations above the LLOQ for 79 of 4,609 (1.7%) participants. As oscillating PK is physiologically implausible in studies administering single doses of mAb therapies, these BLQ observations were removed (i.e., considered missing) from the final model (although sensitivity analyses show that these values have no impact on the results).

### Population PK model development

A two-compartmental distribution model with first-order absorption and elimination was chosen to characterize the PK of AZD7442, cilgavimab, and tixagevimab, based on the results of the “interim” population PK analysis (37). Interim population PK parameter estimates are reported in Table S7. Following a graphical exploration of the data, covariate analysis was performed, and the base model was subsequently updated. The final AZD7442 model was used for simulation analyses, and abbreviated model development for tixagevimab and cilgavimab was performed by refining the final AZD7442 model on an as-needed basis.

Population PK analyses were performed using NONMEM nonlinear mixed-effects modeling software (version 7.4.0; Icon Development Solutions, Ellicott City, MD, USA). Initially, the same structural model as previously developed was considered. As log-transformed serum concentrations were modeled, covariate-parameters relationships were implemented as additive terms in the log-transformed domain of the respective parameter. The previous “interim” model implemented body weight-based allometric scaling with fixed exponents on the following parameters: CL, Vc, Q, and Vp, with 0.75 for rates and 1.0 for volumes. A full covariate modeling approach was employed (38), emphasizing parameter estimation rather than stepwise hypothesis testing. The covariate modeling was aimed at confirming the lack of influence of covariates that were not included in the final model. Lack of influence was defined as a covariate that was not statistically significant and/or not considered potentially clinically relevant. A covariate on a model parameter was defined as not potentially clinically relevant if the absolute relative parameter change induced by this covariate at either the 5th or 95th percentile of a continuous covariate in the data set (or at specific levels for a categorical covariate) was < 20% of the typical value.

#### Model evaluation

Model adequacy and decisions on increasing model complexity were assessed based on the data and goodness-of-fit criteria, including visual inspection of diagnostic scatter plots (e.g., observed versus predicted concentrations), successful convergence of minimization routine, plausibility and precision of the parameter estimates, and Bayesian information criteria. Visual predictive checks were generated by simulation of 500 replicates of the analysis data set to demonstrate the adequacy of the model in describing the observed data across all studies and groups, and thus provide model validation.

### Simulation-based analyses

Simulation-based assessment of the clinical importance of retained covariates was performed by deriving exposure metrics for various covariate combinations of interest in the final AZD7442 model. A typical participant was defined based on the demographic summary (i.e., median value for continuous covariates and most prevalent category for categorical covariates). The typical participant was simulated 1,000–10,000 times, accounting for intra-individual variability and based on a simulated single IM dose of AZD7442 600 mg. Simulations were repeated for several covariate combinations. C_max_ and AUC_0-inf_ were determined from these simulations, and medians and 90% prediction intervals were reported.

## Data Availability

Data underlying the findings described in this manuscript may be requested in accordance with AstraZeneca’s data sharing policy described at https://astrazenecagrouptrials.pharmacm.com/ST/Submission/Disclosure. AstraZeneca Group of Companies allows researchers to submit a request to access anonymized subject-level clinical data, aggregate clinical or genomics data (when available), and anonymized clinical study reports through the Vivli web-based data request platform.

## SUPPLEMENTAL MATERIAL

Supplemental tables and figures aac.01587-23-s0001.docx Tables S1 to S7 and Figures S1 and S2.

ASM does not own the copyrights to Supplemental Material that may be linked to, or accessed through, an article. The authors have granted ASM a non-exclusive, world-wide license to publish the Supplemental Material files. Please contact the corresponding author directly for reuse.
