# Supplementary material for: Accelerating therapeutics development during a pandemic: population pharmacokinetics of the long-acting antibody combination AZD7442 (tixagevimab/cilgavimab) in the prophylaxis and treatment of COVID-19
Source: Antimicrob Agents Chemother. 2024 Mar 27;68(5):e01587-23. doi: 10.1128/aac.01587-23 (PMC11064475; doi:10.1128/aac.01587-23)
Supplement: Supplemental tables and figures — Tables S1 to S7 and Figures S1 and S2. [file aac.01587-23-s0001.docx]

Supplementary Materials

Accelerating Therapeutics Development During a Pandemic: Population Pharmacokinetics of the Long-Acting Antibody Combination AZD7442 (Tixagevimab/Cilgavimab) in the Prophylaxis and Treatment of COVID-19

Lindsay E. Clegg,^a^*# Oleg Stepanov,^b^* Henning Schmidt,^c^ Weifeng Tang,^a^
Huixia Zhang,^a^ Chris Webber,^d^ Taylor S. Cohen,^e^ Mark T. Esser,^e^ and Mats Någård^a^

*Co-first authors: both contributed substantially to the presented analyses. LEC is listed first as she led preparation of the first draft of the manuscript. #Corresponding author.

^a^Clinical Pharmacology and Quantitative Pharmacology, Clinical Pharmacology & Safety Sciences, R&D, AstraZeneca, Gaithersburg, MD, USA

^b^Clinical Pharmacology and Quantitative Pharmacology, Clinical Pharmacology & Safety Sciences, R&D, AstraZeneca Cambridge, UK

^c^IntiQuan GmBH, Basel, Switzerland

^d^Clinical Development, Vaccines and Immune Therapies, BioPharmaceuticals R&D, AstraZeneca, Cambridge, UK

^e^Vaccines & Immune Therapies, BioPharmaceuticals R&D, AstraZeneca, Gaithersburg, MD, USA

**Correspondence to:** Lindsay E. Clegg ([lindsay.clegg1@astrazeneca.com](mailto:lindsay.clegg1@astrazeneca.com))

TABLE S1. Baseline demographics and characteristics of the included population.

| **Characteristic** | **Total^a^ (N = 4,940)** |
| --- | --- |
| Mean age, years (SD) [range] | 50.5 (15.8) [18.0–98.0] |
| Age ≤65 years, n (%) | 4,069 (82.4) |
| Age >65 years, n (%) | 871 (17.6) |
| Female, n (%) | 2,317 (46.9) |
| Race, n (%) |  |
| White | 3,494 (70.7) |
| Black | 668 (13.5) |
| Asian | 431 (8.7) |
| Other / not reported / unknown | 347 (7.0) |
| Ethnicity |  |
| Not Hispanic or Latino | 3,529 (71.4) |
| Hispanic or Latino | 1,223 (24.8) |
| Not reported/unknown | 188 (3.8) |
| Mean height, cm (SD) [range] | 169 (10.1) [122.0–204.0] |
| Mean weight, kg (SD) [range] | 83.9 (21.3) [36.0–216.0] |
| Mean BMI, kg/m^2^ (SD) [range] | 29.2 (6.7) [13.6–72.6] |
| BMI <30 kg/m^2^, n (%) | 2,981 (60.3) |
| ≥30 kg/m^2^, n (%) | 1,959 (39.7) |
| **Medical history** |  |
| Received COVID-19 vaccination^b^, n (%) | 2,293 (46.4) |
| Immunocompromised, n (%) | 39 (0.8) |
| Hematological malignancies or cancer or receiving chemotherapy, n (%) |  |
| Current | 91 (1.8) |
| History | 293 (5.9) |
| Chronic kidney disease, n (%) | 220 (4.5) |
| Chronic liver disease, n (%) | 203 (4.1) |
| Chronic obstructive pulmonary disease, n (%) | 254 (5.1) |
| Cardiovascular disease (hypertension, serious heart conditions), n (%) | 1,645 (33.3) |
| Diabetes, n (%) | 638 (12.9) |
| **Laboratory parameters** |  |
| Mean bilirubin, mg/dL (SD) [range] | 0.43 (0.3) [0.2–3.2] |
| Mean albumin, g/L (SD) [range] | 45.8 (3.4) [26.0–59.0] |
| Mean alanine aminotransferase, U/L (SD) [range] | 24.5 (20.4) [4.0–422.0] |
| Mean aspartate aminotransferase, U/L (SD) [range] | 23.6 (16.1) [4.0–460.0] |
| Mean creatinine clearance, mL/min (SD) [range] | 115 (46.3) [4.3–414.0] |
| Mean eGFR, mL/min/1.73 m^2^ (SD) [range] | 87.7 (25.3) [2.4–386.0]^c^ |
| **Administration and study details** |  |
| Route of administration, n (%) |  |
| IV | 342 (6.9) |
| IM | 4,598 (93.1) |
| Participants by study, n (%) |  |
| Global Phase I | 50 (1.0) |
| PROVENT | 3,288 (66.6) |
| STORM CHASER | 722 (14.6) |
| Japanese Phase I | 30 (0.6) |
| Chinese Phase I | 49 (1.0) |
| Chinese Phase II | 202 (4.1) |
| TACKLE | 442 (9.0) |
| ACTIV-2 | 157 (3.2) |
| Participant in Phase I studies, n (%) | 129 (2.6) |
| Participant in treatment study, n (%) | 599 (12.1) |

^a^Total column shown based on all included studies; bCOVID-19 vaccination administered any time before or during the trial; cMissing values for 23 participants.
BMI, body mass index; BP, blood pressure; COVID, coronavirus disease 2019; eGFR, estimated glomerular filtration rate.

TABLE S2. Base and final AZD7442 population PK model structures.

| **Base model** | Linear two-compartmental distribution.  Linear elimination from the central compartment.  Zero-order administration of IV doses into the central compartment.  First-order absorption of IM doses into the central compartment with an associated FIM and a first-order absorption rate parameter ka.  All fixed effects were estimated.  Random effects were estimated for parameters CL, Vc, Vp, and ka.  No random effects were considered on FIM and Q.  Correlation between the random effects of ka, CL, and Vc was considered.  Separate additive residual error models for log transformed IV and IM data were used. |
| --- | --- |
| **Covariates included in the base model** | Body weight at baseline on CL, Vc, Q, and Vp with fixed allometric exponents (0.75 for rates and 1.0 for volumes). |
| **Final covariate model** | Body weight at baseline on CL, Vc, Q, and Vp with fixed allometric exponents (0.75 for rates and 1.0 for volumes).  Male sex on ka.  Age category on ka.  BMI category on ka.  Diabetes on ka and CL.  Black race on Vc.  Study ACTIV-2 on FIM. It should be noted that the covariate ACTIV-2 in this context is used to distinguish the site of IM administrations. In study ACTIV-2 the site of IM administration was thigh muscle, while in all other studies it was the gluteal region. |

BMI, body mass index; CL, clearance; FIM, absolute intramuscular bioavailability; IM, intramuscular; IV, intravenous; ka, first-order absorption rate parameter; Q, inter-compartmental clearance; Vc, central volume of distribution; Vp, peripheral volume of distribution.

TABLE S3. Additional detail on population PK parameter estimates for the final AZD7442 model.

| **Parameter** | **Value** | **RSE** | **Shrinkage** | **Comment** | | |
| --- | --- | --- | --- | --- | --- | --- |
| **Typical parameters** | | | | |  |  |
| **First-order absorption rate (ka)** | 0.11 | 2.46% | - | (1/days) First-order IM absorption rate parameter | | |
| **Clearance (CL)** | 0.0504 | 1.83% | - | (L/days) Clearance | | |
| **Central volume of distribution (Vc)** | 3.36 | 2.34% | - | (L) Volume of central compartment | | |
| **Absolute intramuscular bioavailability (FIM)** | 0.671 | 1.68% | - | (fraction) Absolute IM bioavailability | | |
| **Inter-compartmental clearance (Q)** | 0.395 | 1.71% | - | (L/days) Inter-compartmental clearance | | |
| **Peripheral volume of distribution (Vp)** | 1.83 | 2.08% | - | (L) Volume of peripheral compartment | | |
| **Inter-individual variability** | | | | |  |  |
| **ka CV%** | 59.89% | 1.39% | 22.3% | LogNormal | | |
| **CL CV%** | 42.64% | 0.979% | 9.4% | LogNormal | | |
| **Vc CV%** | 39.79% | 1.79% | 14.9% | LogNormal | | |
| **FIM CV%** | 0% (FIX) | - | - | - | | |
| **Q CV%** | 0% (FIX) | - | - | - | | |
| **Vp CV%** | 35.88% | 2.93% | 45.5% | LogNormal | | |
| **Correlation of random effects** | | | | | |  |
| **corr(ka,CL)** | -0.387 | 4.07% | - | Correlation coefficient | | |
| **corr(ka,Vc)** | -0.689 | 2.82% | - | Correlation coefficient | | |
| **corr(CL,Vc)** | 0.588 | 1.95% | - | Correlation coefficient | | |
| **Parameter-Covariate relationships** | | | | | |  |
| **beta_ka(SEXM_1)** | 0.543 | 4.54% | - | Sex Male on ka | | |
| **beta_ka(AGECAT_1)** | -0.325 | 9.7% | - | Age category > 65 years on ka | | |
| **beta_ka(BMICAT_1)** | -0.254 | 9.94% | - | BMI category ≥ 30 kg/m2 on ka | | |
| **beta_ka(DIAB_1)** | -0.318 | 11.1% | - | Diabetes Yes on ka | | |
| **beta_CL(BWT)** | 0.75 (FIX) | - | - | Baseline weight in kg on CL (centered around: 70 kg) | | |
| **beta_CL(DIAB_1)** | 0.198 | 8.07% | - | Diabetes Yes on CL | | |
| **beta_Vc(BWT)** | 1 (FIX) | - | - | Baseline weight in kg on Vc (centered around: 70 kg) | | |
| **beta_Vc(RACEB_1)** | -0.251 | 6.91% | - | Black Race Yes on Vc | | |
| **beta_Q(BWT)** | 0.75 (FIX) | - | - | Baseline weight in kg on Q (centered around: 70 kg) | | |
| **beta_Vp(BWT)** | 1 (FIX) | - | - | Baseline weight in kg on Vp (centered around: 70 kg) | | |
| **beta_FIM(ACTIV2_1)** | 0.416 | 7.08% | - | ACTIV-2 Yes on FIM | | |
| **Residual Variability** | | | | | |  |
| **error_ADD1** | 0.24 | 0.216% | 19.8%^a^ | Additive Error (log(μg/mL)) - IM data | | |
| **error_ADD2** | 0.104 | 0.339% | 18.4%^a^ | Additive Error (log(μg/mL)) - IV data | | |
| **Objective function** | -22509 | - | - | - | | |
| **AIC** | -22465 | - | - | - | | |
| **BIC** | -22280 | - | - | - | | |

^a^Epsilon shrinkage (records with missing dependent variable and censored records not considered).
AIC, Akaike information criterion; BIC, Bayesian information criterion; CL, clearance; CV%, percent coefficient of variation; FIM, absolute intramuscular bioavailability; IMP, importance sampling; ka, first order absorption rate constant; Q, inter-compartmental clearance; RSE, relative standard error; Vc, central volume of distribution; Vp, peripheral volume of distribution.

TABLE S4. Additional detail on population PK parameter estimates for the final tixagevimab model.

| **Parameter** | **Value** | **RSE** | **Shrinkage** | **Comment** |
| --- | --- | --- | --- | --- |
| **Typical parameters** | | | | |
| **First-order absorption rate (ka)** | 0.119 | 2.75% | - | (1/days) First-order IM absorption rate parameter |
| **Clearance (CL)** | 0.0515 | 2.37% | - | (L/days) Clearance |
| **Central volume of distribution (Vc)** | 3.52 | 3.47% | - | (L) Volume of central compartment |
| **Absolute intramuscular bioavailability (FIM)** | 0.657 | 2.27% | - | (fraction) Absolute IM bioavailability |
| **Inter-compartmental clearance (Q)** | 0.485 | 2.48% | - | (L/days) Inter-compartmental clearance |
| **Peripheral volume of distribution (Vp)** | 1.82 | 1.74% | - | (L) Volume of peripheral compartment |
| **Inter-individual variability** | | | | |
| **ka CV%** | 77.12% | 1.06% | 28.1% | LogNormal |
| **CL CV%** | 44.08% | 0.863% | 6.4% | LogNormal |
| **Vc CV%** | 52.17% | 1.61% | 13.1% | LogNormal |
| **FIM CV%** | 0% (FIX) | - | - | - |
| **Q CV%** | 0% (FIX) | - | - | - |
| **Vp CV%** | 24.49% | 5.05% | 60.1% | LogNormal |
| **Correlation of random effects** | | | | |
| **corr(ka,CL)** | -0.442 | 3.1% | - | Correlation coefficient |
| **corr(ka,Vc)** | -0.546 | 2.83% | - | Correlation coefficient |
| **corr(CL,Vc)** | 0.764 | 0.915% | - | Correlation coefficient |
| **Parameter-Covariate relationships** | | | | |
| **beta_ka(SEXM_1)** | 0.57 | 4.78% | - | Sex Male on ka |
| **beta_ka(AGECAT_1)** | -0.392 | 8.79% | - | Age category > 65 years on ka |
| **beta_ka(BMICAT_1)** | -0.188 | 14.9% | - | BMI category ≥ 30 kg/m2 on ka |
| **beta_ka(DIAB_1)** | -0.27 | 14.6% | - | Diabetes Yes on ka |
| **beta_CL(BWT)** | 0.75 (FIX) | - | - | Baseline weight in kg on CL (centered around: 70 kg) |
| **beta_CL(DIAB_1)** | 0.149 | 9.37% | - | Diabetes Yes on CL |
| **beta_Vc(BWT)** | 1 (FIX) | - | - | Baseline weight in kg on Vc (centered around: 70 kg) |
| **beta_Vc(RACEB_1)** | -0.26 | 5.94% | - | Black Race Yes on Vc |
| **beta_Q(BWT)** | 0.75 (FIX) | - | - | Baseline weight in kg on Q (centered around: 70 kg) |
| **beta_Vp(BWT)** | 1 (FIX) | - | - | Baseline weight in kg on Vp (centered around: 70 kg) |
| **beta_FIM(ACTIV2_1)** | 0.414 | 8.49% | - | ACTIV-2 Yes on FIM |
| **Residual Variability** | | | | |
| **error_ADD1** | 0.24 | 0.17% | 21.3%^a^ | Additive Error (log(μg/mL)) - IM data |
| **error_ADD2** | 0.119 | 0.422% | 18.1% ^a^ | Additive Error (log(μg/mL)) - IV data |
| **Objective function** | -25204 | - | - | - |
| **AIC** | -25160 | - | - | - |
| **BIC** | -24975 | - | - | - |

^a^Epsilon shrinkage (records with missing dependent variable and censored records not considered).
AIC, Akaike information criterion; BIC, Bayesian information criterion; CL, clearance; corr, correlation; CV%, percent coefficient of variation; FIM, absolute intramuscular bioavailability; IM, intramuscular; IMP, importance sampling; IV, intravenous; ka, first order absorption rate constant; Q, inter-compartmental clearance; RSE, relative standard error; Vc, central volume of distribution; Vp, peripheral volume of distribution.

TABLE S5. Additional detail on population PK parameter estimates for the final cilgavimab model.

| **Parameter** | **Value** | **RSE** | **Shrinkage** | **Comment** |
| --- | --- | --- | --- | --- |
| **Typical parameters** | | | | |
| **First-order absorption rate (ka)** | 0.122 | 2.89% | - | (1/days) First-order IM absorption rate parameter |
| **Clearance (CL)** | 0.0456 | 1.77% | - | (L/days) Clearance |
| **Central volume of distribution (Vc)** | 3.17 | 2.21% | - | (L) Volume of central compartment |
| **Absolute intramuscular bioavailability (FIM)** | 0.615 | 1.66% | - | (fraction) Absolute IM bioavailability |
| **Inter-compartmental clearance (Q)** | 0.432 | 1.99% | - | (L/days) Inter-compartmental clearance |
| **Peripheral volume of distribution (Vp)** | 1.77 | 2.23% | - | (L) Volume of peripheral compartment |
| **Inter-individual variability** | | | | |
| **ka CV%** | 78.56% | 1.17% | 20.2% | LogNormal |
| **CL CV%** | 40.81% | 0.901% | 7.2% | LogNormal |
| **Vc CV%** | 38% | 1.95% | 15.9% | LogNormal |
| **FIM CV%** | 0% (FIX) | - | - | - |
| **Q CV%** | 0% (FIX) | - | - | - |
| **Vp CV%** | 36.85% | 2.85% | 42.5% | LogNormal |
| **Correlation of random effects** | | | | |
| **corr(ka,CL)** | -0.496 | 3.06% | - | Correlation coefficient |
| **corr(ka,Vc)** | -0.835 | 2.68% | - | Correlation coefficient |
| **corr(CL,Vc)** | 0.697 | 1.33% | - | Correlation coefficient |
| **Parameter-Covariate relationships** | | | | |
| **beta_ka(SEXM_1)** | 0.632 | 4.62% | - | Sex Male on ka |
| **beta_ka(AGECAT_1)** | -0.443 | 8.32% | - | Age category > 65 years on ka |
| **beta_ka(BMICAT_1)** | -0.217 | 13.9% | - | BMI category ≥ 30 kg/m2 on ka |
| **beta_ka(DIAB_1)** | -0.262 | 16.3% | - | Diabetes Yes on ka |
| **beta_CL(BWT)** | 0.75 (FIX) | - | - | Baseline weight in kg on CL (centered around: 70 kg) |
| **beta_CL(DIAB_1)** | 0.167 | 8.81% | - | Diabetes Yes on CL |
| **beta_Vc(BWT)** | 1 (FIX) | - | - | Baseline weight in kg on Vc (centered around: 70 kg) |
| **beta_Vc(RACEB_1)** | -0.242 | 6.78% | - | Black Race Yes on Vc |
| **beta_Q(BWT)** | 0.75 (FIX) | - | - | Baseline weight in kg on Q (centered around: 70 kg) |
| **beta_Vp(BWT)** | 1 (FIX) | - | - | Baseline weight in kg on Vp (centered around: 70 kg) |
| **beta_FIM(ACTIV2_1)** | 0.378 | 7.94% | - | ACTIV-2 Yes on FIM |
| **Residual Variability** | | | | |
| **error_ADD1** | 0.272 | 0.196% | 18.1%^a^ | Additive Error (log(μg/mL)) - IM data |
| **error_ADD2** | 0.108 | 0.344% | 19.3%^a^ | Additive Error (log(μg/mL)) - IV data |
| **Objective function** | -23746 | - | - | - |
| **AIC** | -23702 | - | - | - |
| **BIC** | -23517 | - | - | - |

^a^Epsilon shrinkage (records with missing dependent variable and censored records not considered).
AIC, Akaike information criterion; BIC, Bayesian information criterion; CL, clearance; corr, correlation; CV%, percent coefficient of variation; FIM, absolute intramuscular bioavailability; IM, intramuscular; IMP, importance sampling; IV, intravenous; ka, first order absorption rate constant; Q, inter-compartmental clearance; RSE, relative standard error; Vc, central volume of distribution; Vp, peripheral volume of distribution.

TABLE S6. Description of studies included in the pooled population PK analysis.

| **Study description (title,  NCT number)** | **Dose of AZD7442 and PK sampling times** | **Participants/ observations in  PK dataset, N^a^** | **Study population** |
| --- | --- | --- | --- |
| Global Phase I (NCT04507256) | Single dose: 300 mg IM, 300 mg IV (seq), 1,000 mg IV (seq), 3,000 mg IV (seq), 3,000 mg IV (co-administration)^b^  PK sample collection: at 50% of dose administered (IV only), EOI (IV only), 8 hours (IV only), and 3, 5, 7, 14, 30, 60, 90, 150, 210, 270, and 360 days post-dose | 50/836 | Healthy adults |
| Phase III PrEP (PROVENT; NCT04625725) | 300 mg IM  PK samples collected 7, 28, 57, 91, 182, and 365 days post-dose (Day 456 optional)  Some participants received a second dose of AZD7442 before day 365 as part of a substudy | 3,461/24,630 | Adults with increased risk of inadequate response to COVID-19 vaccination or increased risk of exposure to SARS-CoV-2 |
| Phase III post-exposure prophylaxis (STORM CHASER; NCT04625972) | Single dose: 300 mg IM  PK samples collected 7, 28, 57, 91, 182, and 365 days post-dose (Day 456 optional) | 749/5,745 | Adults with exposure to SARS-CoV-2 (within 8 days of dosing) |
| Phase III outpatient treatment (TACKLE; NCT04723394) | Single dose: 600 mg IM  PK samples collected on Day 1, 2, 5, 14, 28, 84, 168, and 365 post-dose (Day 456 optional) | 452/3,296 | Adults with mild-to-moderate COVID-19 |
| Phase II outpatient treatment (ACTIV-2; NCT04518410) | Single dose: 600 mg IM, 300 mg IV  PK samples collected at EOI (IV only) and 3, 7, 14, 28, 84, and 168 days post-dose | 164/1,145 | Adults with COVID-19 |
| Japanese Phase I (NCT04896541) | Single dose: 300 mg IM, 600 mg IM, 300 mg IV, 1,000 mg IV  PK samples collected at EOI (IV only), 8 hours, and 1, 3, 5, 7, 14, 30, 60, 90, 150, 210, 270, and 360 days  post-dose | 30/454 | Healthy Japanese adults |
| Chinese Phase I (NCT05437289) | Single dose: 300 mg IM, 600 mg IM, 300 mg IV, 600 mg IV  PK samples collected at EOI (IV only), 8 hours, and 1, 3, 5, 7, 14, 30, 60, 90, 180, 270, and 360 days post-dose | 49/587 | Healthy Chinese adults |
| Chinese Phase II (NCT05184062) | Single dose: 600 mg IV  PK samples collected at EOI, 7, 30, 60, 90, 180, 270, and 360 days post-dose | 202/1,387 | Chinese adults |

^a^Numbers correspond to the PK dataset before exclusions (as described in the Methods section); ^b^AZD7442 IV was administered either by sequential administration involving 2 sequential infusions of tixagevimab then cilgavimab, or coadministration of a single infusion containing both tixagevimab and cilgavimab.
COVID-19, coronavirus disease 2019; EOI, end of infusion; IM, intramuscular; IV, intravenous; PK, pharmacokinetic; PrEP, pre-exposure prophylaxis; seq, sequential.

TABLE S7. Population PK parameter estimates (95% CI) for AZD7442, cilgavimab, and tixagevimab based on previous analysis of interim data from the PROVENT, STORM CHASER and TACKLE studies and final data from the Phase I first-in-human study of AZD7442.

| **Parameter** | **Unit** | **Cilgavimab** | **Tixagevimab** | **AZD7442** |
| --- | --- | --- | --- | --- |
| Median half-life^a^ (95% CI) | Days | 84.4179  (70.61759–146.48903) | 88.8277  (72.99582–157.33544) | 90.6112  (72.46954–151.18043) |
| CL (RSE%) [95% CI] | L/day | 0.0412 (1.23)  [0.0402–0.0422] | 0.0405 (1.09)  [0.0396–0.0414] | 0.0443 (1.40)  [0.0430–0.0455] |
| Vc (RSE%) [95% CI] | L | 2.48 (4.22) [2.27–2.68] | 2.72 (4.33) [2.49–2.96] | 3.62 (3.47) [3.37–3.87] |
| Q (RSE%) [95% CI] | L/day | 0.595 (3.03)  [0.560–0.630] | 0.588 (3.25)  [0.550–0.625] | 0.614 (2.62)  [0.582–0.645] |
| Vp (RSE%) [95% CI] | L | 2.57 (1.40) [2.50–2.64] | 2.64 (1.83) [2.55–2.74] | 2.60 (1.98) [2.50–2.70] |
| KA (RSE%) [95% CI] | 1/day | 0.106 (3.11)  [0.0999–0.113] | 0.109 (3.59)  [0.102–0.117] | 0.125 (3.22)  [0.117–0.133] |
| F1 (RSE%) [95% CI] | – | 0.593 (0.951)  [0.582–0.604] | 0.617 (0.327)  [0.613–0.621] | 0.669 (0.822)  [0.659–0.680] |
| Additive error (RSE%) [95% CI] | µg/mL | 0.359 (8.02)  [0.302–0.415] | 1.04 (8.92)  [0.858–1.22] | 3.39 (2.25)  [3.24–3.54] |
| Proportional error (RSE%) [95% CI] | – | 0.153 (2.24)  [0.146–0.159] | 0.157 (3.81)  [0.145–0.169] | 0.0968 (4.41)  [0.0885–0.105] |
| Sex on Vc (RSE%) [95% CI] | – | –0.346 (8.91)  [–0.286 to –0.407] | –0.362 (8.56)  [–0.302 to –0.423] | –0.369 (7.07)  [–0.318 to –0.421] |
| Sex on KA (RSE%) [95% CI] | – | 0.481 (11.7)  [0.371–0.591] | 0.579 (11.7)  [0.447–0.712] | 0.449 (12.3)  [0.341–0.558] |
| Age on KA (RSE%) [95% CI] | – | –0.265 (10.5)  [–0.211 to –0.320] | –0.297 (9.79) [–0.240 to –0.354] | –0.275 (10.1)  [–0.220 to –0.329] |
| DIAB on Vc (RSE%) [95% CI] | – | 0.578 (18.3)  [0.371–0.785] | 0.399 (22.8)  [0.220–0.577] | 0.434 (18.6)  [0.275–0.593] |
| DIAB on CL (RSE%) [95% CI] | – | 0.283 (14.0)  [0.205–0.361] | 0.223 (18.9)  [0.140–0.305] | 0.199 (23.4)  [0.107–0.290] |
| IIV CL (RSE%) [95% CI] | %CV | 29.3 (4.49) [26.7–31.8] | 21.3 (4.79) [19.3–23.3] | 20.9 (5.67) [18.6–23.2] |
| IIV CL Vc^b^ (RSE%) [95% CI] | – | 0.885 (2.75)  [0.837–0.932] | 0.836 (3.70)  [0.776–0.897] | 0.809 (4.06)  [0.744–0.873] |
| IIV Vc (RSE%) [95% CI] | %CV | 116 (2.90)  [109–122] | 100 (3.52)  [93.3–107.1] | 82.3 (3.07) [77.3–87.2] |
| IIV Vp (RSE%) [95% CI] | %CV | Not estimated | 6.29 (31.8) [2.37–10.2] | 10.3 (30.8) [4.09–16.6] |
| IIV KA (RSE%) [95% CI] | %CV | 54.5 (4.16) [50.1–59.0] | 54.9 (5.22) [49.3–60.5] | 47.5 (4.81) [43.0–52.0] |

^a^Terminal half-life was derived using micro-constants K12, K21, Kel, Vc, and Vp, and is presented as median and 5^th^ and 95^th^ percentiles.

^b^Estimate of the covariance between CL and Vc. Correlation (CL-Vc) calculated as follows: Covariance (CL-Vc) / Square root (Variance [CL] * Variance [Vc]) * 100.

%CV, percent coefficient of variation; CI, confidence interval; CL, clearance; DIAB, diabetes; F1, absolute bioavailability; IIV, inter-individual variability; KA, absorption rate constant; K12, 1st order distribution rate constant from central to peripheral compartment; K21, 1st order distribution rate constant from peripheral to central compartment; Kel, 1st order elimination rate constant; PopPK, population pharmacokinetic; Q, inter-compartmental clearance; RSE(%), relative standard error; V2, central volume of distribution; V3, peripheral volume of distribution.

FIG S1. Goodness-of-fit plots (observation vs. population and individual predictions) for (A) IM data and (B) IV data.

**
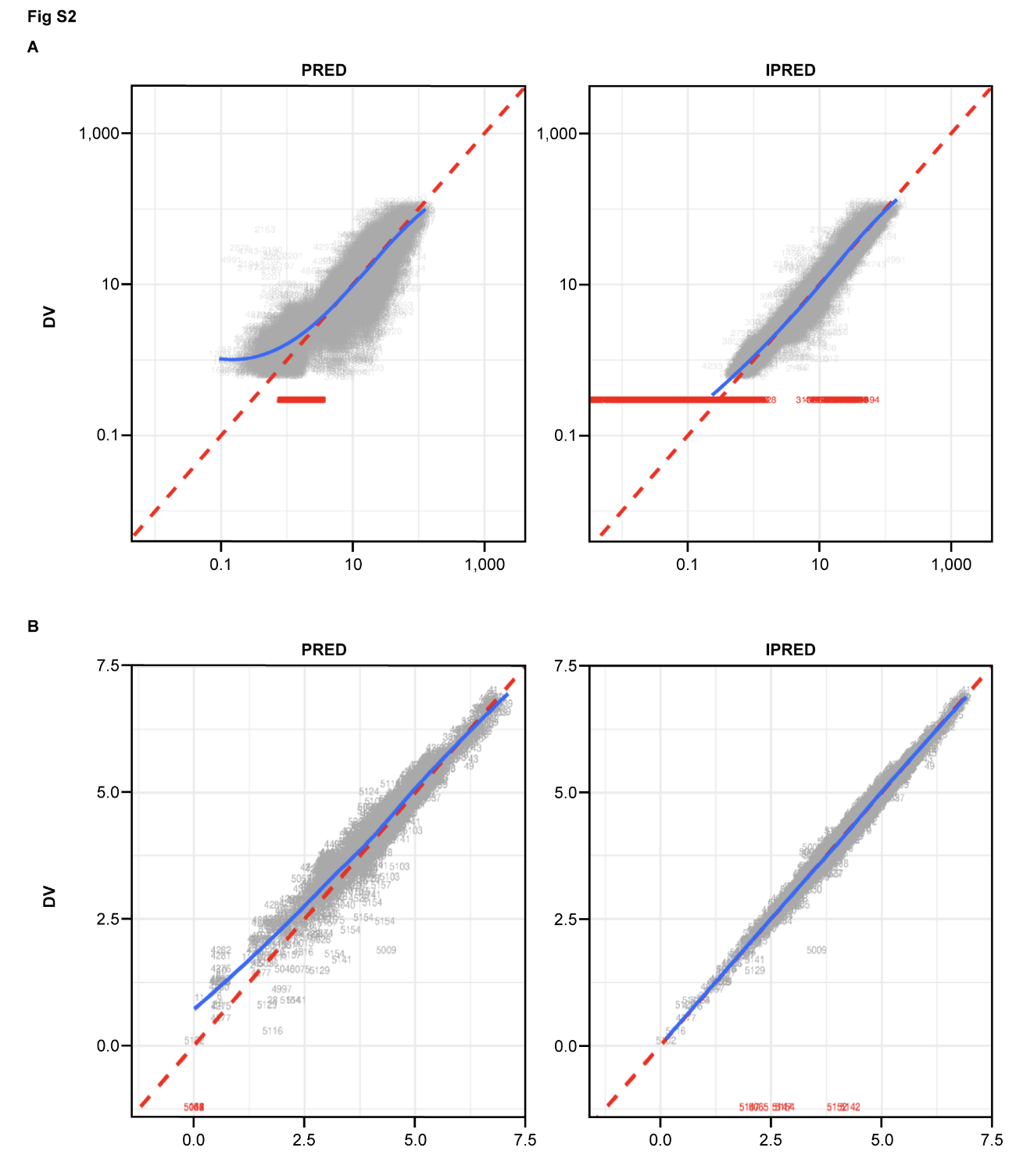
**

Goodness-of-fit plot for IM data log-transformed and zoomed in to focus on non-BLQ values.
DV, dependent variable; IV, intravenous; IPRED, individual prediction; PRED, population prediction.

FIG S2. Visual predictive checks of final models for cilgavimab (A) and tixagevimab (B) IM data, stratified by dose level.

**
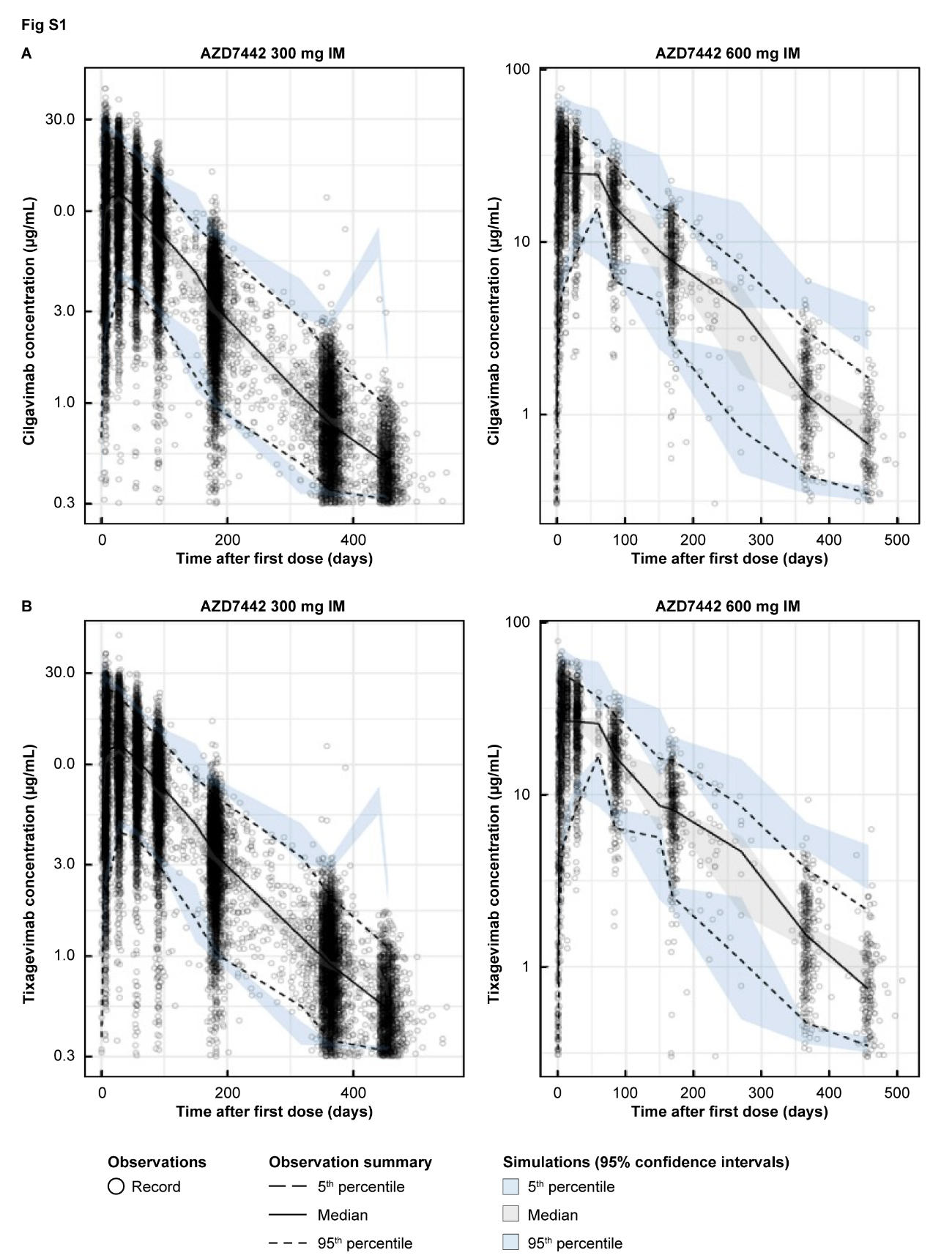
**

DV versus (I)PRED Diagnostics IM Data (log) – AZD7442 Final Model – zoomed in to focus on datapoints rather than BLQ values.
BLQ, below the limit of quantitation; DV, dependent variable; PRED, population prediction; IPRED, individual prediction.
